# Hospital Festivals and Meetings

**Published:** 1895-08-10

**Authors:** 


### HOSPITAL FESTIVALS AND MEETINGS.

### DEMONSTRATION OF PHYSICAL EDUCATION.

A very interesting demonstration and "grand military assault-at-arms" was given on Wednesday, July 24th, at the Portman Rooms, Baker Street, by the students of the People's Palace Gymnasia, under the direction of Mr. H. H. Burdett (member and examiner of the Royal College of Physical Education), and members of the Army Gymnastic Staff of Aldershot, by permission of Colonel G. M. Fox (instructor of gymnasia). It was on the occasion of the opening of Mr. Burdett's Gymnasium and School of Training for Children, 24, Baker Street, W., and the chair was taken by the Earl of Meath, whose interest in the promotion of physical training is well known. The displays of strength and skill on the parallel bars, in boxing, foil fencing, and cavalry sword exercises by Sergeant-Major W. Palmer, Staff Sergeants Singleton, Wallace, and others were splendid, and it was only to be regretted that they were not enjoyed by a larger audience. Mr. Burdett's students went through very creditable bar-bell and dumb-bell exercise, and Mr. Burdett himself executed marvellous feats of swordsmanship in the lemon and glove cutting line, finishing with the remarkable performance of cutting the carcase of a sheep in two with a single cut. The People's Palace students had an interest all their own. The paramount necessity for wholesome physical training to counteract the unwholesome conditions under which the lives of large numbers of working Londoners are passed, so peculiarly injurious to physical development, does not obtain anything like the general recognition it deserves, and any opportunity for bringing the subject before the public must be welcomed, tending, as it should do, to make those who are large employers of labour realise their duties in this respect towards their workmen, to whom every facility and encouragement should be given to induce the taking up in their leisure hours of a training which will develop and strengthen their physical forces. Mr. Burdett's pupils undoubtedly do him every credit, and it is most satisfactory to note the evident popularity of his classes, and that the number of members increases largely every year.

### THE YARROW CONVALESCENT HOME FOR CHILDREN.

### A New Departure.

The opening of the convalescent home at Broadstairs, on the 31st ult., was attended with special interest. Mr. Yarrow's munificent offering to sick children is intended to benefit classes who, though needing the immense benefit which a short seaside residence affords after illness, have hitherto been unprovided for, public generosity having been turned towards helping the very poor. There are now convalescent homes all over the United Kingdom ready to welcome these, and, therefore, none can begrudge the children of the professional man, the poor minister, the city clerk, and the better class of artisan, the benefits they frequently are so really in need of, and which they yet could not accept without some payment in return. To such children the home should prove a veritable paradise, for nothing has been left undone to secure their comfort, and no thought or trouble spared to select the most salubrious spot possible of attainment.

The thoughtful kindness and perfect organisation of the arrangements on the occasion of the opening on the 31st augurs well for the future administration of the home. A special train conveyed Mr. Yarrow's guests from London, who all assembled in the entrance hall of the home for the informal opening ceremony. A short prayer was offered up, a hymn was sung, and then Mrs. Yarrow, who first suggested the admirable scheme to Mr. Yarrow, unlocked the door and declared the institution open. After an excellent and most tastefully arranged lunch, toasts were drunk and short speeches made. Mr. Yarrow, in response to a speech proposing his health, explained the objects of the Home. So completely did he suppress any allusion to his part in the undertaking that the uninitiated might have departed unaware of the fact that foundation and endowment were solely due to his munificence. We cannot refrain from an expression of admiration at the manner in which the founder suppressed the "ego" on an occasion where a little self-congratulation and pride might certainly have been called forth.

The arrangements by which the guests viewed the establishment in different companies under the conductorship of stewards prevented any undue crowding, and rendered a complete inspection possible. The appearance of the building, both back and front, is excellent. It is built of red brick with a great deal of white woodwork introduced, which gives a light and pleasing effect. The frontage towards the sea is especially inviting, with its long covered balcony parallel with the first floor. Within, the eye of the visitor is at once charmed with the air of brightness and comfort noticeable throughout. A soft buff colour predominates, which harmonises with the creamy white of the woodwork. Every corridor and room is decorated with delightful pictures, mostly depicting scenes in child-life. In the day-rooms comfortable couches and chairs invite weary little frames, and a good supply of toys and books are ready for wet days. In the wards the cots and beds are of excellent pattern, and nice little locker appertain to each. The lavatories, where stand rows of white Rufford baths and basins fitted with brass hot and cold water taps, the linen cupboards, kitchens and larders, all called forth admiration from the matrons of the party. In fact, had we gone with the unpleasant intention of searching for weak spots we might have departed disappointed. Everywhere, however, there was evidence that not only had the most warm-hearted generosity been at work, but that both skill and knowledge had been exercised throughout. Mr. Yarrow secured the assistance of the best expert opinion available, and was most ably seconded by his architects, Messrs. Davis and Emanuel, and the contractors, Messrs. Colls and Son.

The delightful home, which can accommodate 100 children, has already a promise of little inmates. Domestic and nursing staffs are already installed—the latter under the guidance of Miss Power, late of the Great Ormond Street Children's Hospital. We hope before very long to give a technical description of the buildings, together with the plans.

